# ECRI Institute Guidelines Trust

**DOI:** 10.5195/jmla.2019.693

**Published:** 2019-07-01

**Authors:** Jessica Shira Sender

**Affiliations:** Librarian for the College of Nursing, Michigan State University, Lansing, MI, jsender@msu.edu

## INTRODUCTION

The ECRI Guidelines Trust is a new database intended to replace the Agency for Healthcare Research and Quality (AHRQ) National Guidelines Clearinghouse. The AHRQ National Guidelines Clearinghouse was shut down in July 2018 after the AHRQ did not receive funding to continue its work [1]. The National Guidelines Clearinghouse was originally created in 1997 in partnership with the American Medical Association and the American Association of Health Plans, and it existed for over twenty years [2].

With the demise of the AHRQ National Guidelines Clearinghouse, there was a need for this type of resource to be available to faculty, students, doctors, nurses, and the general public. As Tiffany Leung wrote, “As a primary care and urgent care physician and as a clinical informatician, I know first-hand the value of guidelines in practice and the opportunities they offer in patient care and in research” [3]. The ECRI Institute was the prime contractor for the National Guidelines Clearinghouse, so the transition to host a guidelines database in their suite of products was a natural next step. The ECRI Guidelines Trust is an appropriate resource for those looking to find current clinical practice guidelines.

## EVALUATING GUIDELINES FOR INCLUSION

Getting guidelines into the ECRI Guidelines Trust is rigorous and requires adherence to the Institute of Medicine (IOM) definition of clinical practice guidelines [4].

The ECRI Guidelines Trust describes itself as “a publicly available web-based repository of objective, evidence-based clinical practice guideline content” [5]. The ECRI Guidelines Trust outlines the inclusion criteria on their website, and only after a guideline meets all criteria and has been approved by reviewers will it be included in the ECRI Guidelines Trust database. Additionally, if a guideline meets some, but not all of the criteria, it will undergo additional review for possible inclusion as a link to the full-text original guideline [5, 6]. Guidelines also must be approved by the guideline developer in order to be included in the ECRI Guidelines Trust.

ECRI Guidelines Trust outlines the inclusion criteria for guidelines as follows [6]:

1. Guidelines must be available in English, can be free or for a fee, and must have been published within the last five years
2. Guidelines include recommendation statements providing guidance on patient care
3. Guidelines must be produced by a medical specialty association, professional medical society, or other relevant clinical practice guideline development organization
4. Guidelines must be based on a verifiable systematic review of evidence that includes:
  - Search strategy
    - databases searched
    - search terms
    - specific time-period covered by literature search
  - Study selection
    - number of studies identified
    - number of studies included after inclusion/exclusion criteria
    - summary of inclusion/exclusion criteria
  - Evidence analysis

Each included guideline also has a Transparency and Rigor Using Standards of Trustworthiness (TRUST) Scorecard [4]. A TRUST Scorecard is based on the AHRQ National Guideline Clearinghouse Extent Adherence to Trustworthy Standards (NEATS) and “assesses clinical practice guidelines against the Institute of Medicine (IOM) standards for trustworthy guidelines” [4]. NEATS was the assessment developed under ECRI’s former contract with the AHRQ National Guidelines Clearinghouse [4]. The NEATS assessments were publicly available, so ECRI is using NEATS to inform the TRUST Scorecard that they developed.

## SEARCHING THE ECRI GUIDELINES TRUST

Searches on the ECRI Guidelines Trust can be entered in a single search box; however, there is no advanced search option at this time. The content is well organized, and users will appreciate the straightforward navigation. Results contain information about the guideline’s origination, which is a useful feature.

There are options to limit a search by year, organization, patient population, clinical area, and intervention. While this feature will be quite helpful in the future, the ECRI Guidelines Trust is still a relatively small database, so the ability to narrow in this way was only marginally helpful at the time of this review.

Many guidelines have a “guideline brief,” which includes an overview and breakdown of the content in each entry, allowing users to jump to different relevant sections of the document. The brief includes a basic overview of the guideline as well as the patient population that it is intended to serve, but also more in-depth information like the methodology of developing the guideline. For example, in the Guideline on Hand Hygiene from the Association of PeriOperative Registered Nurses (AORN), the methodology outlines the systematic review conducted by a librarian as well as the inclusion and exclusion criteria used, and the evaluation of articles reviewed for the systematic review. Each guideline also has associated benefits and risks, funders, and related content including patient education, when available. These resources can be used in a classroom setting as well and may be particularly helpful when showing students how systematic reviews inform evidence-based practice and care.

The ECRI Guidelines Trust is a free resource, although an account must be created to save searches and previously browsed topics.

## AREAS FOR DEVELOPMENT

In this reviewer’s opinion, one of the biggest drawbacks is that the ECRI Guidelines Trust is not as robust and content-rich as the National Guidelines Clearinghouse was. This is to be expected since the ECRI Guidelines Trust has had to essentially rebuild the National Guidelines Clearinghouse from the ground up. Additionally, the ECRI Guidelines Trust is continually adding resources to their database.

The many different acronyms, scorecard terminology, and inclusion criteria can be confusing to users, and perhaps additional and more streamlined search aids will be included in the future.

Lastly, some guidelines link directly out to the original guideline and do not have a brief or a scorecard. As previously mentioned, if a guideline only meets one to three of the inclusion criteria, ECRI may instead include a link to the full-text original [6]. Users then have to refer back to these requirements to determine the appropriateness and reliability of the inclusion. Again, additional search aids could help reduce this ambiguity for searchers.

## CONCLUSION

The ECRI Guidelines Trust is a reliable and much needed resource, particularly after the closure of the National Guidelines Clearinghouse. Clinical guidelines can be quite difficult to find, and the ability to go to a single resource instead of individual journal or organizational websites is an advantage for faculty, students, and practitioners alike.

For librarians, this resource helps navigate the landscape following the loss of the National Guidelines Clearinghouse and is a good source to refer patrons to. The ECRI Guidelines Trust is also a helpful addition for those who are conducting systematic reviews and large literature reviews, particularly when they are looking for grey literature or guidelines that are not published in traditional peer-reviewed journals. Additionally, the rigor that guidelines must go through to be included in the ECRI Guidelines Trust gives librarians a resource that they can recommend with confidence.

Ultimately, this is a resource that is appropriate for librarians, faculty, and clinicians in a wide variety of settings from hospitals to academic libraries. Future content development and the expansion of resources in the ECRI Guidelines Trust will make this an asset to any collection.
